# Generative Pre-Trained Transformers (GPT) and Space Health: A Potential Frontier in Astronaut Health During Exploration Missions

**DOI:** 10.1017/S1049023X23005848

**Published:** 2023-08

**Authors:** Ethan Waisberg, Joshua Ong, Mouayad Masalkhi, Nasif Zaman, Sharif Amit Kamran, Prithul Sarker, Andrew G. Lee, Alireza Tavakkoli

**Affiliations:** 1. University College Dublin School of Medicine, Belfield, Dublin, Ireland; 2. Michigan Medicine, University of Michigan, Ann Arbor, Michigan USA; 3. Human-Machine Perception Laboratory, Department of Computer Science and Engineering, University of Nevada - Reno, Reno, Nevada USA; 4. Center for Space Medicine, Baylor College of Medicine, Houston, Texas USA; 5. Department of Ophthalmology, Blanton Eye Institute, Houston Methodist Hospital, Houston, Texas USA; 6. The Houston Methodist Research Institute, Houston Methodist Hospital, Houston, Texas USA; 7. Departments of Ophthalmology, Neurology, and Neurosurgery, Weill Cornell Medicine, New York, New York USA; 8. Department of Ophthalmology, University of Texas Medical Branch, Galveston, Texas USA; 9. University of Texas MD Anderson Cancer Center, Houston, Texas USA; 10. Texas A&M College of Medicine, Bryan, Texas USA; 11. Department of Ophthalmology, The University of Iowa Hospitals and Clinics, Iowa City, Iowa USA

**Keywords:** artificial intelligence, generative pre-trained transformer, spaceflight associated neuro-ocular syndrome, space medicine

## Abstract

In anticipation of space exploration where astronauts are traveling away from Earth, and for longer durations with an increasing communication lag, artificial intelligence (AI) frameworks such as large language learning models (LLMs) that can be trained on Earth can provide real-time answers. This emerging technology may be helpful for acute medical emergencies, particularly in austere and distant space environments. In this manuscript, we provide an overview of generative pre-trained transformer (GPT) technology, a rapidly emerging AI technology, and implications, considerations, and limitations of such technology for space health.

## Introduction

From the droid C-3PO in *Star Wars* (1977) to the robot bartender in *Passengers* (2016), a myriad of science fiction movies have envisioned an automated, machine-based program with the artificial intelligence (AI) ability to react to novel scenarios and provide real-life answers. Several of these science fiction movies also include automated programs that provide medical guidance, particularly when no living medical expert is around. With the advent of AI, we are approaching a time where certain AI frameworks may make this science fiction concept into a closer reality. In anticipation of space exploration where astronauts are traveling away from Earth, and for longer durations with an increasing communication lag, these AI frameworks can be trained on Earth to provide real-time answers. This emerging technology may be helpful for acute medical emergencies, particularly in austere and distant space environments. In this manuscript, we provide an overview of generative pre-trained transformer (GPT) technology, a rapidly emerging AI technology, and implications, considerations, and limitations of such technology for space health.

Very recently, ChatGPT (Open AI; San Francisco, California USA) has quickly acquired popularity after its introduction in late November 2022, thanks to its thorough responses and human-like writing skills.^1^ Chat GPT architecture, or ChatGPT, a large language model (LLM), involves a transformer neural network which generates human-like text from deep-learning techniques, natural language processing (NLP), and self-attention mechanisms, among many other algorithms.^2^ As such, the network is composed of several linked layers, or “transformer blocks,” which analyze the user input and offer an output suggestion.^2^

Using AI, ChatGPT has revealed promising results. For example, its ability to generate images based on patient descriptions of complex neuro-ophthalmic visual phenomena.^3^ Remarkably, AI technologies are rapidly evolving and are expected to revolutionize the field of medicine. When used appropriately, AI may enhance patient care and make it more secure and effective, with some key applications already implemented in personalized medicine and diagnostic imaging.^4,5^ Medical practice is likely to undergo significant change as a result of the rapid development of AI technology. As these technologies reach clinical use, skills to interpret and use AI in a medical setting will become essential for doctors.

With the recent commercialization of spaceflight, more lay individuals are expected to travel to space in the coming years than ever before. This commercialization of spaceflight also allows a new diverse population to be able to visit space, in contrast to the younger, physically fit, and thoroughly medically screened current population of astronauts. These new space travelers can potentially have multiple comorbidities and have additional polypharmacy concerns. The physiological impacts of spaceflight such as venous fluid shifts, altered vestibulo-ocular function,^6^ and increased levels of hemolysis^7,8^ may affect these individuals more profoundly, and potentially lead to a medical emergency.

**Figure 1.**
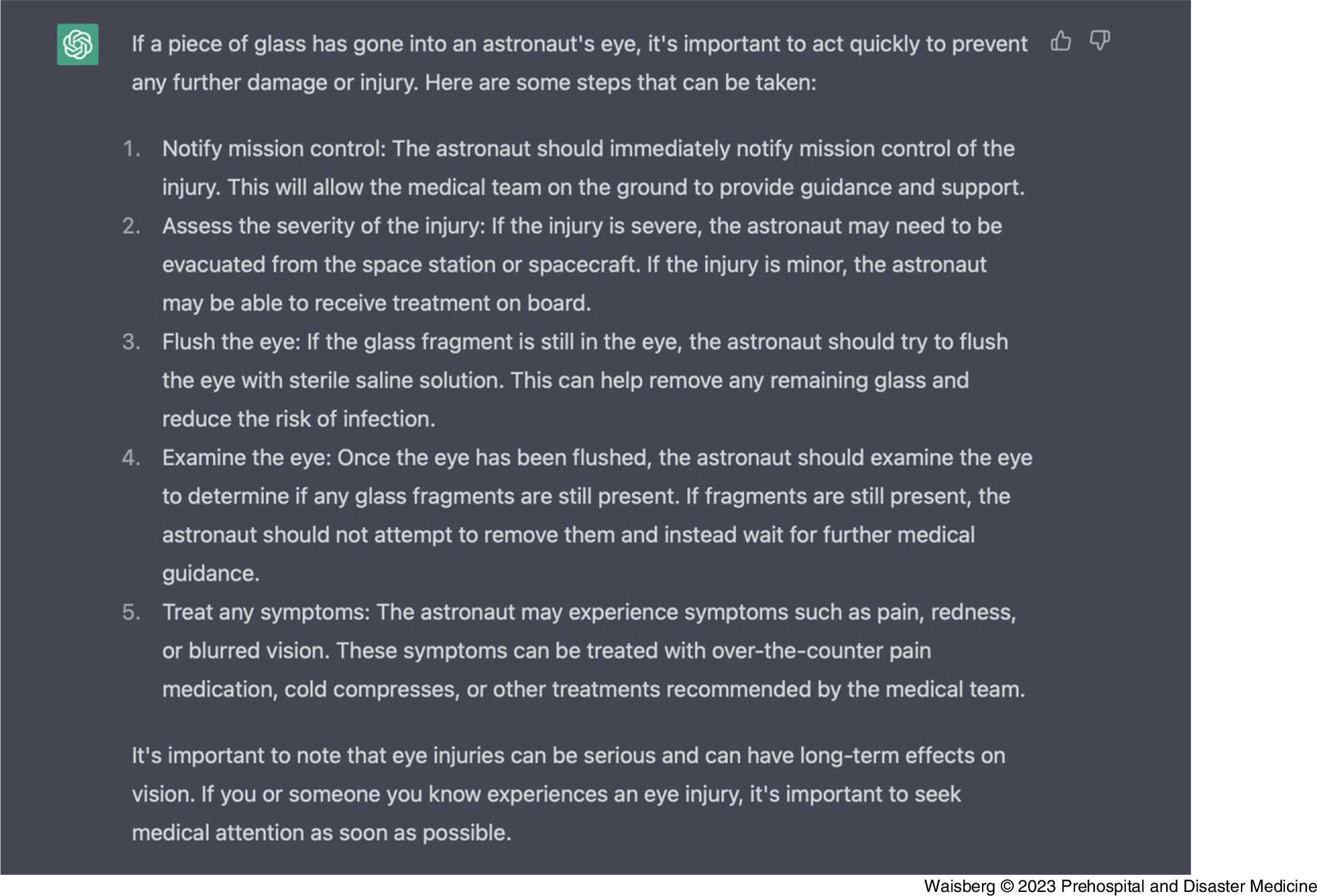
ChatGPT-Generated Response from the Prompt: “Help. A Piece of Glass was Floating and Unexpectedly Went Into an Astronaut’s Eye.” Abbreviation: GPT, generative pre-trained transformer.

To examine the performance of ChatGPT at providing advice during a medical emergency, we asked ChatGPT what to do if glass unexpectedly entered an astronaut’s eye (Figure 1).

**Figure 2.**
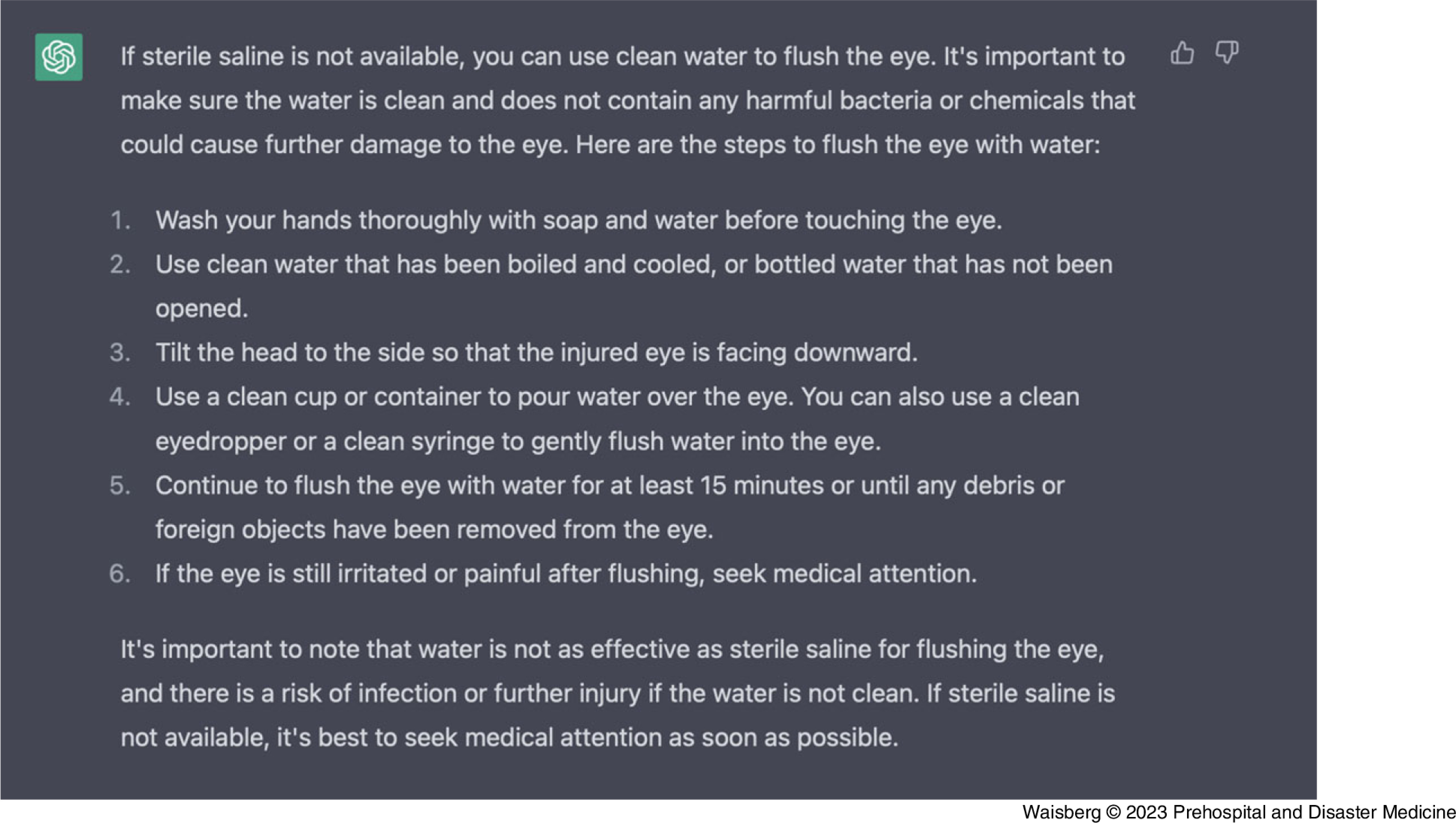
ChatGPT-Generated Response from the Prompt: “We Don’t Have Any Sterile Saline, Are There Any Alternatives?” Abbreviation: GPT, generative pre-trained transformer.

This advice is very effective with first notifying mission control to try and receive medical support, then assessing severity, flushing the eye with sterile saline, and re-examining the eye afterwards and treating any symptoms. To assess ChatGPT’s ability to improvise and use limited materials due to the constraints of being in space, we told ChatGPT that no sterile saline was available (Figure 2).

With this limitation of not using normal saline, ChatGPT recommended using water (that is clean as possible) to flush out the eye instead, and rightfully warned of potential bacterial and chemicals in unclean water which may lead to further ocular damage. ChatGPT also recommended to flush the eye continuously for 15 minutes at least, or until all foreign objects are removed, which is also helpful advice.

**Figure 3.**
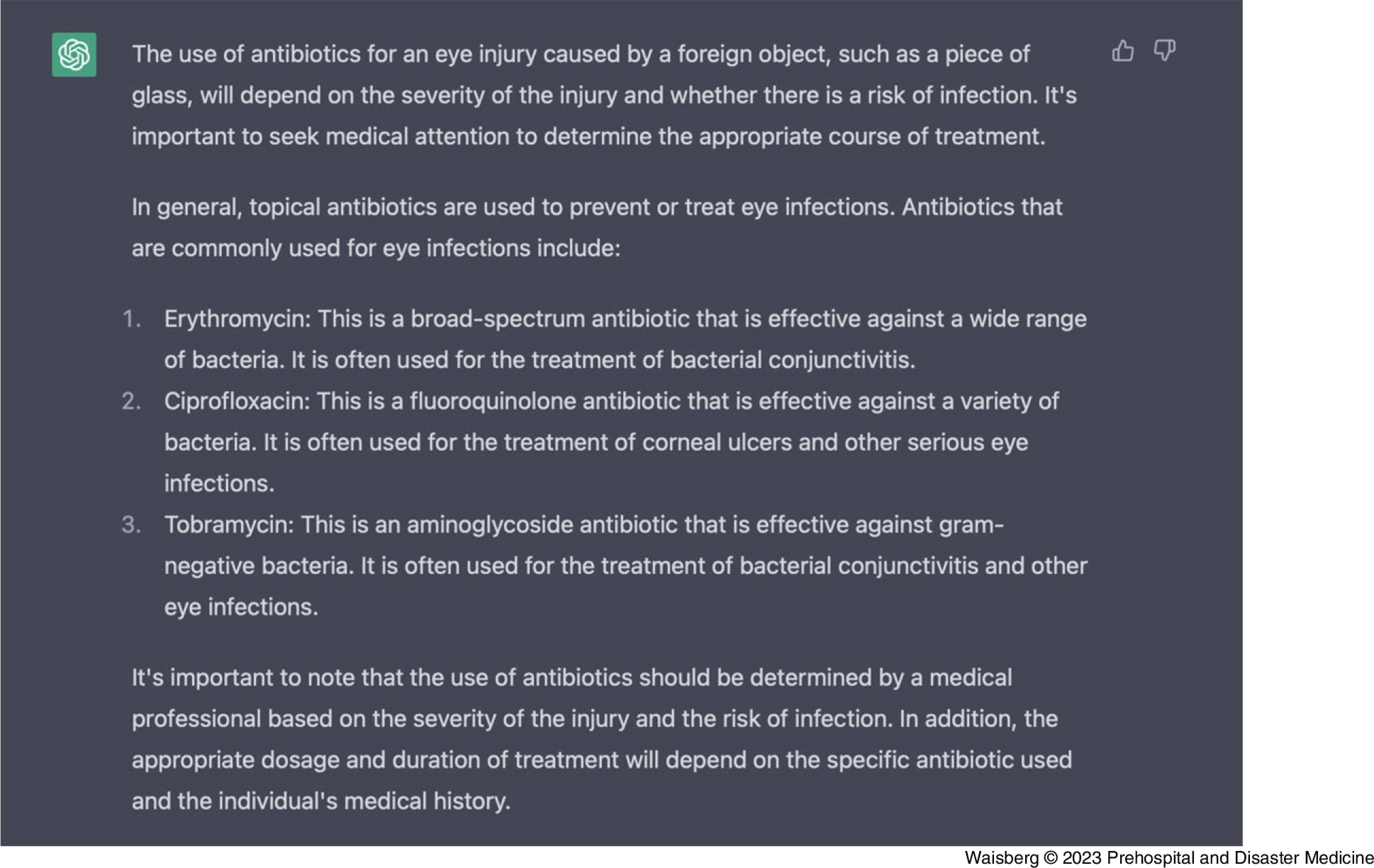
ChatGPT-Generated Response from the Prompt: “What Antibiotics Should I Use?” Abbreviation: GPT, generative pre-trained transformer.

We then asked ChatGPT which antibiotic should be used for this eye injury (Figure 3).

ChatGPT correctly recommended that the usage of antibiotics depends on eye injury severity and the associated risk of infection and how antibiotic choice should be determined by an ophthalmic medical professional. ChatGPT then provided relatively accurate information on the indications of several commonly used topical antibiotics used to treat eye infections, such as: erythromycin, ciprofloxacin, and tobramycin.

**Figure 4.**
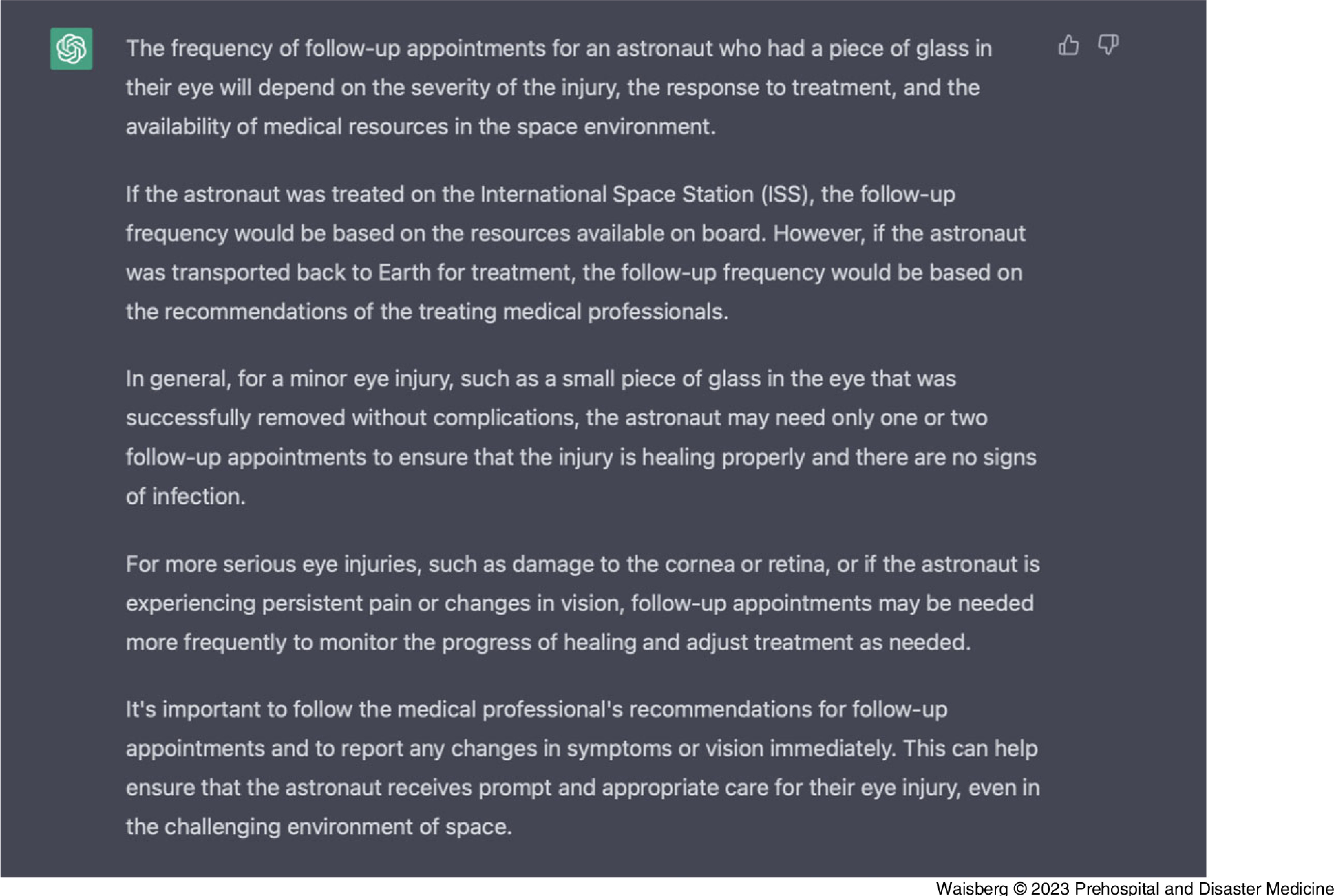
ChatGPT-Generated Response from the Prompt: “How Frequently Should I Follow-Up Once the Astronaut That Had Glass in His Eye is Stabilized?” Abbreviation: GPT, generative pre-trained transformer.

Finally, we asked ChatGPT how much follow-up is required for the astronaut after their condition is stabilized (Figure 4).

ChatGPT correctly identified that the amount of follow-up is dependent on the injury severity, available medical resources in space, and the astronaut’s treatment response. ChatGPT also correctly stated that if the glass was successfully removed without complications, only one or two follow-up appointments are required to ensure there is no signs of infection and to show that healing is occurring. We were also impressed with the safety-netting provided by ChatGPT, that the patient should immediately report back if any new visual changes or other symptoms occur.

Even with a physician in space who has trained for austere environments and the unique challenges of spaceflight, the wide variety of potential medical issues remains broad and can be across nearly any medical specialty. Additionally, during space missions that travel away from Earth, signal delays are expected to occur, delaying communications which may be critical during medical emergencies. ChatGPT and other future LLMs may play a key role in helping astronauts to manage medical emergencies during long-duration spaceflight. ChatGPT can provide recommended solutions to manage medical emergencies in simple language, in an easy-to-follow manner. These rapidly generated recommendations may be useful in an emergency setting with limited expertise.

## Limitations

However, ChatGPT, much like any AI model, has certain limitations. ChatGPT was developed using a substantial body of content (web pages, books, research journals, and other materials); hence, it may offer responses that are entirely or partially similar to previously published texts. Space medicine is a highly specialized specialty that needs a thorough grasp of space physiology and radiation exposure. As a result, ChatGPT may be lacking in domain expertise, resulting in erroneous or irrelevant replies.^9^ It is also possible that the model won’t comprehend the broader scope of a question, which might lead to misunderstandings and errors.^9^ A method to circumvent this may be to train a GPT system specifically on space medicine and austere environment materials. Transfer learning may potentially be employed to address this lack of relevant domain expertise.^10^ While less of a concern for space medicine, ChatGPT self-copying when given a question many times, and a high degree of direct or “word-for-word” plagiarism from internet sources such as Wikipedia (San Francisco, California USA) and LinkedIn (Sunnyvale, California USA) exist.^9^ There are several steps that may be taken to avoid or mitigate these issues. This includes confirming ChatGPT findings by referring to the most recent clinical guidelines/resources and providing more context when presenting queries to or chatting with ChatGPT so that the model can better understand the context of the inquiry.^9^

## Conclusion

All things considered, LLMs like ChatGPT may potentially be useful in emergency settings during exploration spaceflight missions where there is an increasing communication lag to Earth medical specialists. Future research is required to improve the reliability of LLMs and evaluate their efficacy in medical emergencies in space medicine. This research may take in the form of training an LLM solely on austere medicine and space medicine materials. Ultimately, the astronauts are trained professionals to deal with medical emergencies, thus this tool helps to supplement their expertise and training for unanticipated scenarios requiring emergent intervention.
